# Persistent Left Superior Vena Cava Suggested by an Unusual Central Venous Pressure Waveform

**DOI:** 10.1055/s-0040-1708474

**Published:** 2020-04-28

**Authors:** Sujana Dontukurthy, Yoshikazu Yamaguchi, Joseph D. Tobias

**Affiliations:** 1 Department of Anesthesiology and Pain Medicine, Nationwide Children's Hospital, Columbus, Ohio, United States; 2 Department of Anesthesiology, The Ohio State University, Columbus, Ohio, United States

**Keywords:** cardiovascular surgery, myocardial protection, anatomy, congenital heart disease, CHD

## Abstract

**Background** A persistent left superior vena cava (PLSVC) is the most common congenital anomaly of the thoracic venous return.

**Case Description** During atrial septal defect repair, a pulmonary artery (PA) catheter was placed via the left internal jugular vein. Although placement of the PA catheter in the main PA was confirmed by transesophageal echocardiography, the central venous pressure (CVP) waveform was abnormal. Intraoperatively, the PA catheter was seen exiting the coronary sinus with the CVP port within the coronary sinus.

**Conclusions** The diagnosis of PLSVC is discussed and the differential diagnosis of the abnormal “ventricular” pattern of the CVP waveform is reviewed.

## Introduction

Although it is the most common congenital anomaly of the thoracic venous return, a persistent left superior vena cava (PLSVC) has a reported incidence of less than 0.5% in the general population. 1 2 3 4 The incidence is higher (10–12%) in association with other forms of congenital heart disease, most commonly atrial (ASD) and ventricular septal defects. PLSVC results from a failure of obliteration of the left common cardinal vein during embryological development. In the majority (80–90%) of cases, the PLSVC drains the left subclavian and jugular veins into the right atrium via the coronary sinus and is of no hemodynamic consequence. It is often found incidentally during cardiovascular imaging or surgery. In a small percentage of patients, it may drain into the left atrium resulting in a right-to-left shunt with the potential for paradoxical emboli. 5 6

We present an 82-year-old man who presented for coronary artery bypass surgery and repair of an ASD. The presence of a PLSVC was suggested by an abnormal waveform on the central venous pressure (CVP) tracing and confirmed intraoperatively. The diagnosis and implications of PLSVC are discussed and the differential diagnosis of the abnormal pattern of the CVP waveform is reviewed.

## Case Report

**Fig. 1:**
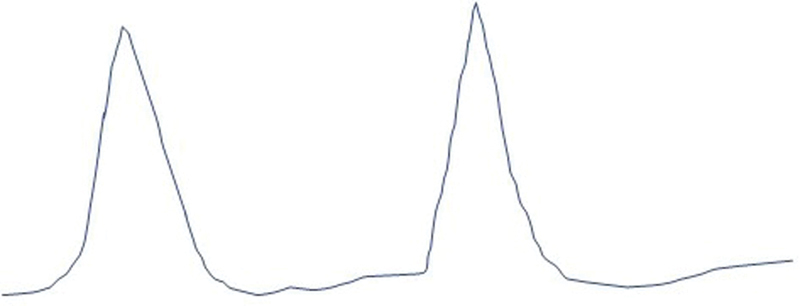
Representation of the abnormal central venous pressure (CVP) waveform noted in our patient.

**Fig. 2:**
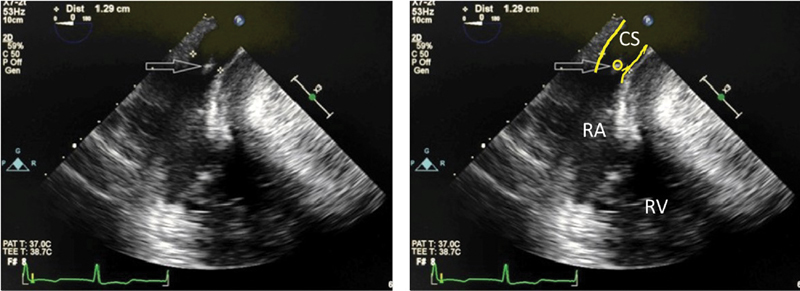
Intraoperative transesophageal echocardiogram (TEE) showing the pulmonary artery (PA) catheter (yellow circle) exiting the coronary sinus (yellow lines) into the right atrium. The coronary sinus diameter was 1.29 cm. CS, coronary sinus; RA, right atrium; RV, right ventricle.

**Fig. 3:**
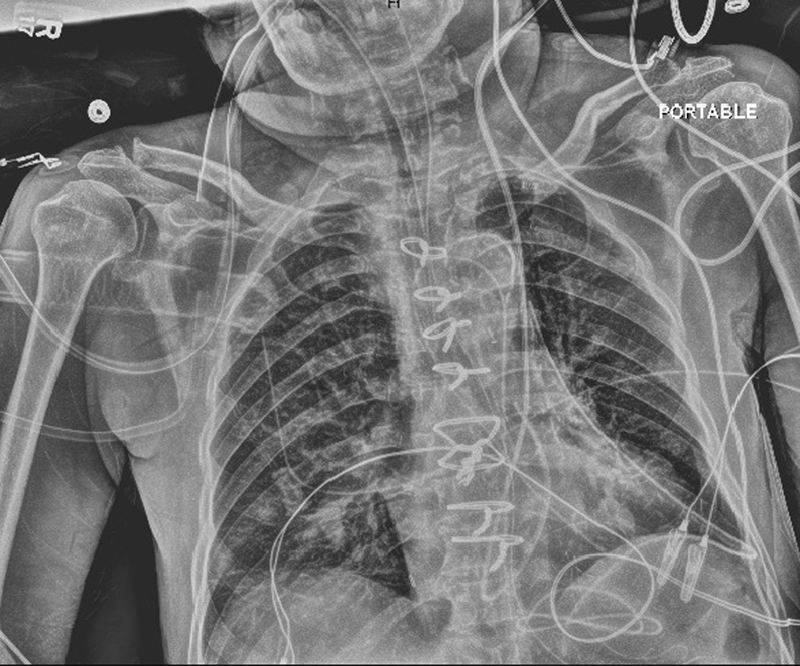
Postoperative chest radiograph demonstrating the abnormal course of the pulmonary artery catheter as it exits the coronary sinus and passes through the tricuspid valve into the right ventricular and the pulmonary artery.

Institutional Review Board approval is not required for presentation of single patient case reports. Verbal consent for publication was obtained from the patient by one of the authors (S.D.). An 82-year-old man with end-stage renal disease requiring hemodialysis, coronary artery disease, secundum type ASD, and mild aortic regurgitation was scheduled for coronary artery bypass surgery and ASD repair. The preoperative echocardiogram showed mild depression of left ventricular function, secundum type ASD, mild right ventricular hypertrophy, and mild aortic regurgitation with no other valvular abnormalities. Preoperative cardiac catheterization revealed coronary artery disease. After the induction of general anesthesia and endotracheal intubation, ultrasound revealed a thrombus in the right internal jugular vein, thought to be due to previous placement of a hemodialysis catheter. Therefore, the left internal jugular vein was cannulated for placement of a pulmonary artery (PA) catheter. The position of the PA catheter in the main PA was confirmed by transesophageal echocardiography (TEE). However, the CVP waveform was noted to be abnormal ( Fig. 1 ). After the initiation of cardiopulmonary bypass, the plan was to administer both antegrade and retrograde cardioplegia due to the presence of aortic regurgitation. The surgeon encountered difficulty with insertion of the retrograde coronary sinus perfusion device into the coronary sinus. Upon opening the right atrium for the ASD repair, the PA catheter was seen exiting the coronary sinus into the right atrium and passing through the tricuspid valve to the right ventricle and the PA. This was confirmed by TEE ( Fig. 2 ). The CVP port was located within the coronary sinus. A PLSVC was noted and subsequently clamped to allow for the effective delivery of retrograde cardioplegia. The remainder of the intraoperative and postoperative courses was uneventful. The postoperative chest radiograph demonstrated the abnormal course of the PA catheter ( Fig. 3 ).

## Discussion

The diagnosis of PLSVC is usually made incidentally during cardiovascular imaging or surgery. During echocardiography, a coronary sinus diameter greater than 1 cm is suggestive of a PLSVC. Other differential diagnosis for a dilated coronary sinus include total or partial anomalous pulmonary venous return with drainage to the coronary sinus, a coronary artery fistula with drainage into the coronary sinus, coronary sinus ASD, right ventricular hypertension, right atrial dysfunction, and severe pulmonary hypertension. The diagnosis of PLSVC may be confirmed by injecting agitated saline into a peripheral vein on the left arm with subsequent visualization of the small bubbles (agitated saline) in the coronary sinus before entering the right atrium. 1 2 3 Other imaging modalities to confirm the diagnosis include contrast venography, computed tomography, and three-dimensional enhanced magnetic resonance venography.

**Fig. 4:**
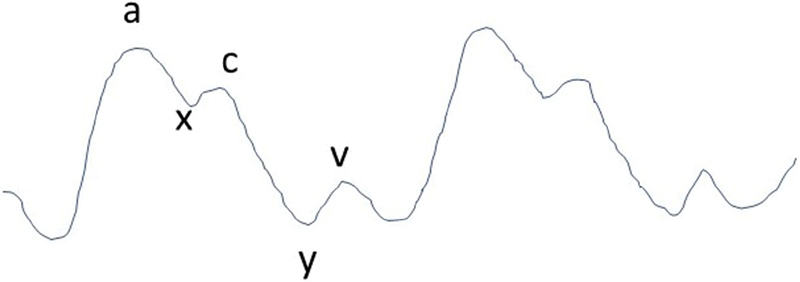
The normal central venous pressure (CVP) waveform containing five components, three peaks (a, c, v) and two descents (x, y), in one cardiac cycle.

The CVP waveform contains three waves or peaks and two descents in one cardiac cycle ( Fig. 4 ). As was noted in our patient, the CVP waveform may be abnormal with a pattern described as a “ventricle pattern or ventricularization” if the opening of the CVP port is in the coronary sinus instead of the right atrium. 5 6 The abnormal pattern or ventricularization results from transmission of pressure from the left ventricle to the coronary sinus. Other causes of the ventricular pattern of the right atrial waveform include severe tricuspid regurgitation, restrictive cardiomyopathy, and the administration of retrograde cardioplegia through a coronary sinus catheter. 5 6

While generally asymptomatic and of no clinical consequences, rare clinical implications related to PLSVC include an abnormal course or malposition of a PA catheter as noted in our patient with the potential for coronary sinus perforation due to inadvertent coronary sinus cannulation. PLSVC has also been associated with an increased risk of cardiac arrhythmias especially atrial fibrillation, paradoxical embolization if there is an associated ASD or drainage into the left atrium, and dilatation of the coronary sinus leading to obstruction of the mitral valve with a predisposition to thrombus formation. 7 8 Additionally, as noted in our patient, retrograde cardioplegia may be ineffective as the cardioplegia solution will be drained into the PLSVC. Clamping the PLSVC is required to achieve effective retrograde cardioplegia. In our patient, the PLSVC was suggested by the presence of a dilated coronary sinus on TEE and the unusual CVP waveform. The PLSVC was confirmed by direct visualization of the surgeon.
